# Novel biomarkers for early detection of HCC in patients with MASLD or ALD

**DOI:** 10.1097/HC9.0000000000000894

**Published:** 2026-01-21

**Authors:** Sneha Deodhar, Mohammad Jarrah, Neehar D. Parikh, Mohammed Al-Hasan, Purva Gopal, Amit G. Singal

**Affiliations:** 1 Department of Internal Medicine, UT Southwestern Medical Center, Dallas, Texas, USA; 2 Department of Medicine, University of Michigan, Ann Arbor, Michigan, USA; 3 Department of Pathology, UT Southwestern Medical Center, Dallas, Texas, USA

**Keywords:** biomarkers, early detection, hepatocellular carcinoma, liver cancer, screening

Hepatocellular carcinoma (HCC) surveillance is associated with improved early detection and survival in patients with cirrhosis.^1^ However, ultrasound plus alpha fetoprotein (AFP) misses one-third of early-stage HCC and is underused in practice.^2^^,^^3^ These limitations have generated interest in alternative imaging and blood-based surveillance strategies.

Osteopontin (OPN) and insulin-like growth factor binding protein 3 (IGFBP3) are multifunctional proteins involved in regulating cell proliferation and tumor progression, with potential roles for early cancer detection.^4^ A meta-analysis reported OPN has a sensitivity of 72% (95% CI 69%–75%) for any-stage HCC, which increases to 81% (95% CI 77%–84%) when combined with AFP.^5^ IGFBP3 levels are lower in HCC versus cirrhosis and may also be a complementary biomarker to AFP and DCP (des-γ-carboxy prothrombin).^6^

However, most studies focused on any-stage HCC, with few evaluating their performance for early-stage detection. Further, most data were derived from populations with a high proportion of viral hepatitis, whereas HCC is increasingly associated with alcohol-associated liver disease (ALD) and metabolic dysfunction–associated steatotic liver disease (MASLD). Liver disease etiology impacts ultrasound and biomarker performance, so data in these populations are critical.^7^^,^^8^ Herein, we evaluated the performance of OPN and IGFBP3 for HCC detection in patients with MASLD or ALD.

We conducted a case–control study among patients with MASLD or ALD cirrhosis from UT Southwestern Medical Center and Parkland Health, recruited between October 2014 and February 2020. The cases were patients with HCC [per AASLD (American Association for the Study of Liver Diseases) criteria], whereas controls had cirrhosis without HCC for >1 year after sample collection. Early-stage HCC was defined as Barcelona Clinic Liver Cancer (BCLC) stage 0/A.^9^ Serum was collected using Early Detection Research Network protocols, processed within 4 hours, and stored at −80 °C without interval thaw and re-freeze. Samples were shipped to Roche for analysis using the Elecsys immunoassay platform, blinded to HCC status.

Diagnostic performance for biomarkers was assessed using the area under the receiver operating characteristic curve (AUROC). Sensitivity and specificity for any-stage and early-stage HCC were assessed at cutoffs per the Youden index (given the lack of validated cutoffs) and at 90% specificity. Diagnostic performance was compared with AFP (thresholds of 20 and 11 ng/mL) using chi-square tests. We evaluated the complementary nature of biomarkers among patients with ultrasound completed within 6 months. Analyses were performed using Stata. The UT Southwestern Medical Center IRB approved the study.

Characteristics of HCC cases (n=71) and cirrhosis controls (n=81) are in Supplemental Table S1, http://links.lww.com/HC9/C234. Cases were older than controls, with median ages of 65 and 55 years, respectively. Liver etiology was evenly distributed between MASLD (52.6%) and ALD (47.4%). Half (52.6%) of patients had Child–Pugh A cirrhosis, with a higher proportion in controls than cases. Most cases (57.7%) had unifocal HCC, and over half (54.9%) had BCLC stage 0/A HCC, with 9.9% BCLC B, 23.9% BCLC C, and 11.3% BCLC D.

Median levels of OPN, IGFBP3, and AFP in cases and controls are illustrated in Figure 1. Table 1 details the performance characteristics of each biomarker for any-stage and early-stage HCC.

**FIGURE 1:**
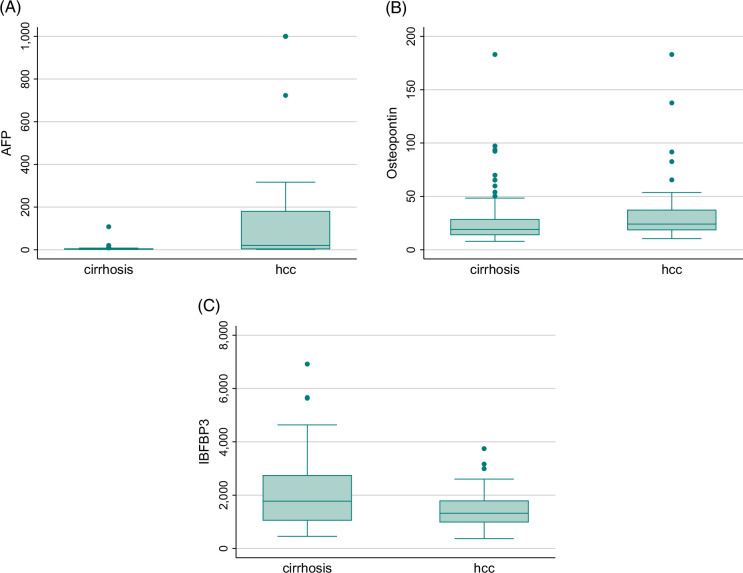
Distribution of AFP, osteopontin, and IGFBP3 for early-stage HCC compared with cirrhosis. Compared to patients with cirrhosis, those with early-stage HCC had significantly higher AFP (*p*<0.001) (A), higher osteopontin (*p*=0.13) (B), and significantly lower IGFBP3 (*p*=0.01) (C). Abbreviations: AFP, alpha fetoprotein; HCC, hepatocellular carcinoma; IGFBP3, insulin-like growth factor binding protein 3.

**TABLE 1:** Performance characteristics of biomarkers for any-stage and early-stage HCC detection

Biomarker	AUROC	Cutoff (ng/mL)	Sensitivity (%)	Specificity (%)
Any-stage HCC				
AFP	0.85 (95% CI 0.78–0.91)	≥20	56.3	98.8
		≥11	63.4	97.5
OPN	0.73 (95% CI 0.65–0.81)	≥23.9	70.4	69.1
		≥50.8	32.4	90
IGFBP3	0.70 (95% CI 0.62–0.79)	≤1479	74.7	63.0
		≤682	16.9	90
Early-stage HCC				
AFP	0.80 (95% CI 0.71–0.90)	≥20	51.3	98.8
		≥11	53.9	97.5
OPN	0.63 (95% CI 0.52–0.73)	≥17.3	84.6	45.7
		≥51.9	15.4	90
IGFBP3	0.63 (95% CI 0.52–0.73)	≤1670	71.8	54.3
		≤679	5.1	90

Abbreviations: AFP, alpha fetoprotein; AUROC, area under the receiver operating characteristic curve; HCC, hepatocellular carcinoma; IGFBP3, insulin-like growth factor binding protein 3; OPN, osteopontin.

For any-stage HCC, OPN, and IGFBP3 had AUROCs of 0.73 (95% CI 0.65–0.81) and 0.70 (95% CI 0.62–0.79), respectively, compared with 0.85 (95% CI 0.78–0.91) for AFP (*p*=0.02 and *p*=0.01, respectively). Using the Youden index, sensitivities and specificities of OPN ≥23.9 ng/mL were 70.4% and 69.1%, and IGFBP3 ≤1479 ng/mL were 74.7% and 63.0%. Sensitivities and specificities were 56.3% and 98.8% for AFP ≥20 ng/mL and 63.4% and 97.5% for AFP ≥11 ng/mL. OPN’s sensitivity did not significantly differ from AFP at both cutoffs (*p*=0.08 and 0.37, respectively), whereas IGFBP3’s sensitivity was significantly higher than AFP ≥20 ng/mL (*p*=0.02) but not AFP ≥11 ng/mL (*p*=0.15). With thresholds defined at 90% specificity, OPN ≥50.8 ng/mL and IGFBP3 ≤682 ng/mL had sensitivities of 32.4% and 16.9%, respectively.

For early-stage HCC, OPN and IGFBP3 had AUROCs of 0.63 (95% CI 0.52–0.73) and 0.63 (95% CI 0.52–0.73), respectively, versus 0.80 (95% CI 0.71–0.90) for AFP (*p*=0.01 for both). Using the Youden index, OPN ≥17.3 ng/mL had a sensitivity and specificity of 84.6% and 45.7%, while IGFBP3 ≤1670 ng/mL had a sensitivity and specificity of 71.8% and 54.3%. Sensitivities were 51.3% for AFP ≥20 and 53.9% for AFP ≥11 ng/mL. AFP sensitivities were lower than OPN (*p*≤0.003 for both) but did not significantly differ from IGFBP3 (*p*=0.06 and 0.10, respectively). With thresholds defined at 90% specificity, OPN ≥51.9 ng/mL and IGFBP3 ≤679 ng/mL had sensitivities of 15.4% and 5.1%, respectively—both significantly lower than AFP at both cutoffs (all *p*<0.001).

Combining either OPN or IGFBP3 at 90% specificity thresholds to AFP ≥20 ng/mL minimally increased sensitivity for early-stage HCC to 53.8%. However, OPN and IGFBP3, using the Youden index, were complementary to AFP. AFP ≥20 ng/mL plus OPN ≥17.3 had a sensitivity and specificity of 92.3% and 45.7% for early-stage HCC. Similarly, AFP ≥20 ng/mL plus IGFBP3 ≥1670 had a sensitivity and specificity of 87.2% and 54.3% for early-stage HCC.

Ultrasound alone sensitivity for early-stage HCC was 52.6%, which increased to 68.4% when combined with AFP ≥20 ng/mL (*p*=0.32) and 94.7% when combined with OPN ≥17.3 ng/mL or IGFBP3 ≤1670 ng/mL (*p*=0.003 for both). Conversely, OPN and IGFBP3 at 90% specificity failed to increase early-stage HCC sensitivity when combined with ultrasound (sensitivity below 60% for both).

Our study adds to the literature by reporting OPN and IGFBP3 performance for early-stage HCC detection in patients with non-viral liver disease. Both had promising sensitivity for early-stage HCC at optimal thresholds, particularly when combined with ultrasound or AFP; however, low specificity estimates would lead to surveillance-related harms. Although patients and providers prioritize surveillance benefits, specificity should still exceed 80% to avoid unacceptable harms.^10^ When specificity was fixed at 90%, both had insufficient sensitivity for early-stage HCC and provided little benefit when added to ultrasound or AFP.

Notably, biomarker performance is overestimated in case–control studies given selection and spectrum biases. Further, results using the Youden index are likely overfit to the study population, so future studies should validate thresholds for biomarker phase 2 and phase 3 studies. Finally, our sample size precluded subgroup analyses by liver disease etiology and severity.

## CONCLUSIONS

OPN and IGFBP3 can achieve high sensitivity for early-stage HCC, but suboptimal specificity may limit their clinical utility.

## Supplementary Material
