# Supplementary material for: Novel biomarkers for early detection of HCC in patients with MASLD or ALD
Source: Hepatol Commun. 2026 Jan 21;10(2):e0894. doi: 10.1097/HC9.0000000000000894 (PMC12826249; doi:10.1097/HC9.0000000000000894)
Supplement: Supplementary file 1 [file hc9-10-e0894-s001.docx]

**Supplemental Table 1.** Characteristics of cases and controls

|  | **HCC cases (n=71)** | **Cirrhosis controls (n=81)** | **p-value** |
| --- | --- | --- | --- |
| **Age (median, IQR)** | 65 (47 – 82) | 55 (38 – 74) | <0.001 |
| **Gender (% male)** | 46 (64.8%) | 40 (49.4%) | 0.07 |
| **Race / Ethnicity**  Non-Hispanic White  Black  Hispanic White | 28 (39.4%)  4 (5.6%)  37 (52.1%) | 36 (44.4%)  1 (1.2%)  39 (48.1%) | 0.32 |
| **BMI (median, IQR)** | 30 (22.3 – 34.9) | 31.3 (20.9 – 49.3) | 0.58 |
| **Liver disease etiology**  MASLD  Alcohol-associated | 38 (53.5%)  33 (46.5%) | 42 (51.9%)  39 (48.1%) | 0.87 |
| **Child-Pugh (% Child A)** | 31 (43.7%) | 50 (64.9%) | 0.01 |
| **BCLC Stage**  Stage 0/A  Stage B  Stage C  Stage D | 39 (54.9%)  7 ( 9.9%)  17 (23.9%)  8 (11.3%) | N/A | N/A |
